# Supplementary material for: Resolving the full spectrum of human genome variation using Linked-Reads
Source: Genome Res. 2019 Apr;29(4):635–45. doi: 10.1101/gr.234443.118 (PMC6442396; doi:10.1101/gr.234443.118)
Supplement: Supplemental Material [file supp_gr.234443.118_Supplemental_longranger-2.2.zip › longranger-2.2.tar.bz2!/lr2.2-public/lib/stan/cmdstan/stan/doc/api/html/double__exponential_8hpp_source.html]

Stan: src/stan/prob/distributions/univariate/continuous/double\_exponential.hpp Source File


|  |  |
| --- | --- |
|  | Stan  2.5.0  probability, sampling & optimization |


- Main Page
- Related Pages
- Namespaces
- Classes
- Files

- File List
- File Members

All Classes Namespaces Files Functions Variables Typedefs Enumerations Enumerator Friends Macros Pages

- src
- stan
- prob
- distributions
- univariate
- continuous

double\_exponential.hpp

Go to the documentation of this file.

1 #ifndef STAN\_\_PROB\_\_DISTRIBUTIONS\_\_DOUBLE\_EXPONENTIAL\_HPP

2 #define STAN\_\_PROB\_\_DISTRIBUTIONS\_\_DOUBLE\_EXPONENTIAL\_HPP

3

4 #include <boost/random/uniform\_01.hpp>

5 #include <boost/random/variate\_generator.hpp>

6

7 #include <stan/agrad/partials\_vari.hpp>

8 #include <stan/math/error\_handling.hpp>

9 #include <stan/math/functions/value\_of.hpp>

10 #include <stan/meta/traits.hpp>

11 #include <stan/prob/constants.hpp>

12 #include <stan/prob/traits.hpp>

13 #include <stan/math/functions/sign.hpp>

14

15 namespace stan {

16

17  namespace prob {

18

19  // DoubleExponential(y|mu,sigma) [sigma > 0]

20  // FIXME: add documentation

21  template <bool propto,

22  typename T\_y, typename T\_loc, typename T\_scale>

23  typename return\_type<T\_y,T\_loc,T\_scale>::type

24  double\_exponential\_log(const T\_y& y,

25  const T\_loc& mu, const T\_scale& sigma) {

26  static const char\* function

27  = "stan::prob::double\_exponential\_log(%1%)";

28

29  using stan::is\_constant\_struct;

30  using stan::math::check\_finite;

31  using stan::math::check\_positive\_finite;

32  using stan::math::check\_consistent\_sizes;

33  using stan::math::value\_of;

34  using stan::prob::include\_summand;

35  using std::log;

36  using std::fabs;

37  using stan::math::sign;

38

39  // check if any vectors are zero length

40  if (!(stan::length(y)

41  && stan::length(mu)

42  && stan::length(sigma)))

43  return 0.0;

44

45  // set up return value accumulator

46  double logp(0.0);

47  check\_finite(function, y, "Random variable", &logp);

48  check\_finite(function, mu, "Location parameter", &logp);

49  check\_positive\_finite(function, sigma, "Scale parameter", &logp);

50  check\_consistent\_sizes(function,y,mu,sigma,

51  "Random variable","Location parameter",

52  "Shape parameter",&logp);

53

54  // check if no variables are involved and prop-to

55  if (!include\_summand<propto,T\_y,T\_loc,T\_scale>::value)

56  return 0.0;

57

58  // set up template expressions wrapping scalars into vector views

59  VectorView<const T\_y> y\_vec(y);

60  VectorView<const T\_loc> mu\_vec(mu);

61  VectorView<const T\_scale> sigma\_vec(sigma);

62  size\_t N = max\_size(y, mu, sigma);

63  agrad::OperandsAndPartials<T\_y,T\_loc,T\_scale> operands\_and\_partials(y, mu, sigma);

64

65  DoubleVectorView<include\_summand<propto,T\_y,T\_loc,T\_scale>::value,is\_vector<T\_scale>::value>

66  inv\_sigma(length(sigma));

67  DoubleVectorView<!is\_constant\_struct<T\_scale>::value,is\_vector<T\_scale>::value>

68  inv\_sigma\_squared(length(sigma));

69  DoubleVectorView<include\_summand<propto,T\_scale>::value,is\_vector<T\_scale>::value>

70  log\_sigma(length(sigma));

71  for (size\_t i = 0; i < length(sigma); i++) {

72  const double sigma\_dbl = value\_of(sigma\_vec[i]);

73  if (include\_summand<propto,T\_y,T\_loc,T\_scale>::value)

74  inv\_sigma[i] = 1.0 / sigma\_dbl;

75  if (include\_summand<propto,T\_scale>::value)

76  log\_sigma[i] = log(value\_of(sigma\_vec[i]));

77  if (!is\_constant\_struct<T\_scale>::value)

78  inv\_sigma\_squared[i] = inv\_sigma[i] \* inv\_sigma[i];

79  }

80

81

82  for (size\_t n = 0; n < N; n++) {

83  const double y\_dbl = value\_of(y\_vec[n]);

84  const double mu\_dbl = value\_of(mu\_vec[n]);

85

86  // reusable subexpressions values

87  const double y\_m\_mu = y\_dbl - mu\_dbl;

88  const double fabs\_y\_m\_mu = fabs(y\_m\_mu);

89

90  // log probability

91  if (include\_summand<propto>::value)

92  logp += NEG\_LOG\_TWO;

93  if (include\_summand<propto,T\_scale>::value)

94  logp -= log\_sigma[n];

95  if (include\_summand<propto,T\_y,T\_loc,T\_scale>::value)

96  logp -= fabs\_y\_m\_mu \* inv\_sigma[n];

97

98  // gradients

99  double sign\_y\_m\_mu\_times\_inv\_sigma(0);

100  if (!is\_constant\_struct<T\_y>::value || !is\_constant\_struct<T\_loc>::value)

101  sign\_y\_m\_mu\_times\_inv\_sigma = sign(y\_m\_mu) \* inv\_sigma[n];

102  if (!is\_constant\_struct<T\_y>::value) {

103  operands\_and\_partials.d\_x1[n] -= sign\_y\_m\_mu\_times\_inv\_sigma;

104  }

105  if (!is\_constant\_struct<T\_loc>::value) {

106  operands\_and\_partials.d\_x2[n] += sign\_y\_m\_mu\_times\_inv\_sigma;

107  }

108  if (!is\_constant\_struct<T\_scale>::value)

109  operands\_and\_partials.d\_x3[n] += -inv\_sigma[n] + fabs\_y\_m\_mu \* inv\_sigma\_squared[n];

110  }

111  return operands\_and\_partials.to\_var(logp);

112  }

113

114

115  template <typename T\_y, typename T\_loc, typename T\_scale>

116  typename return\_type<T\_y,T\_loc,T\_scale>::type

117  double\_exponential\_log(const T\_y& y, const T\_loc& mu,

118  const T\_scale& sigma) {

119  return double\_exponential\_log<false>(y,mu,sigma);

120  }

121

136  template <typename T\_y, typename T\_loc, typename T\_scale>

137  typename return\_type<T\_y,T\_loc,T\_scale>::type

138  double\_exponential\_cdf(const T\_y& y,

139  const T\_loc& mu, const T\_scale& sigma) {

140  static const char\* function

141  = "stan::prob::double\_exponential\_cdf(%1%)";

142

143  // Size checks

144  if ( !( stan::length(y) && stan::length(mu)

145  && stan::length(sigma) ) )

146  return 1.0;

147

148  using stan::math::value\_of;

149  using stan::math::check\_finite;

150  using stan::math::check\_positive\_finite;

151  using stan::math::check\_not\_nan;

152  using boost::math::tools::promote\_args;

153

154  double cdf(1.0);

155  check\_not\_nan(function, y, "Random variable", &cdf);

156  check\_finite(function, mu, "Location parameter", &cdf);

157  check\_positive\_finite(function, sigma, "Scale parameter", &cdf);

158

159  agrad::OperandsAndPartials<T\_y, T\_loc, T\_scale>

160  operands\_and\_partials(y, mu, sigma);

161

162  VectorView<const T\_y> y\_vec(y);

163  VectorView<const T\_loc> mu\_vec(mu);

164  VectorView<const T\_scale> sigma\_vec(sigma);

165  size\_t N = max\_size(y, mu, sigma);

166

167  //cdf

168  for (size\_t n = 0; n < N; n++) {

169  const double y\_dbl = value\_of(y\_vec[n]);

170  const double mu\_dbl = value\_of(mu\_vec[n]);

171  const double sigma\_dbl = value\_of(sigma\_vec[n]);

172  const double scaled\_diff = (y\_dbl - mu\_dbl) / (sigma\_dbl);

173  const double exp\_scaled\_diff = exp(scaled\_diff);

174

175  if(y\_dbl < mu\_dbl) {

176  cdf \*= exp\_scaled\_diff \* 0.5;

177  }

178  else

179  cdf \*= 1.0 - 0.5 / exp\_scaled\_diff;

180  }

181

182  //gradients

183  for (size\_t n = 0; n < N; n++) {

184  const double y\_dbl = value\_of(y\_vec[n]);

185  const double mu\_dbl = value\_of(mu\_vec[n]);

186  const double sigma\_dbl = value\_of(sigma\_vec[n]);

187  const double scaled\_diff = (y\_dbl - mu\_dbl) / sigma\_dbl;

188  const double exp\_scaled\_diff = exp(scaled\_diff);

189  const double inv\_sigma = 1.0 / sigma\_dbl;

190

191  if(y\_dbl < mu\_dbl) {

192  if (!is\_constant\_struct<T\_y>::value)

193  operands\_and\_partials.d\_x1[n] += inv\_sigma \* cdf;

194  if (!is\_constant\_struct<T\_loc>::value)

195  operands\_and\_partials.d\_x2[n] -= inv\_sigma \* cdf;

196  if (!is\_constant\_struct<T\_scale>::value)

197  operands\_and\_partials.d\_x3[n] -= scaled\_diff \* inv\_sigma \* cdf;

198  }

199  else {

200  const double rep\_deriv = cdf \* inv\_sigma / (2.0 \* exp\_scaled\_diff

201  - 1.0);

202  if (!is\_constant\_struct<T\_y>::value)

203  operands\_and\_partials.d\_x1[n] += rep\_deriv;

204  if (!is\_constant\_struct<T\_loc>::value)

205  operands\_and\_partials.d\_x2[n] -= rep\_deriv;

206  if (!is\_constant\_struct<T\_scale>::value)

207  operands\_and\_partials.d\_x3[n] -= rep\_deriv \* scaled\_diff;

208  }

209  }

210  return operands\_and\_partials.to\_var(cdf);

211  }

212

213  template <typename T\_y, typename T\_loc, typename T\_scale>

214  typename return\_type<T\_y,T\_loc,T\_scale>::type

215  double\_exponential\_cdf\_log(const T\_y& y, const T\_loc& mu,

216  const T\_scale& sigma) {

217  static const char\* function

218  = "stan::prob::double\_exponential\_cdf\_log(%1%)";

219

220  using stan::math::check\_finite;

221  using stan::math::check\_not\_nan;

222  using stan::math::check\_positive\_finite;

223  using stan::math::check\_consistent\_sizes;

224  using stan::math::value\_of;

225

226  double cdf\_log(0.0);

227

228  // check if any vectors are zero length

229  if (!(stan::length(y)

230  && stan::length(mu)

231  && stan::length(sigma)))

232  return cdf\_log;

233

234  check\_not\_nan(function, y, "Random variable", &cdf\_log);

235  check\_finite(function, mu, "Location parameter", &cdf\_log);

236  check\_positive\_finite(function, sigma, "Scale parameter", &cdf\_log);

237  check\_consistent\_sizes(function, y, mu, sigma,

238  "Random variable", "Location parameter",

239  "Scale Parameter", &cdf\_log);

240

241  using std::log;

242  using std::exp;

243

244  agrad::OperandsAndPartials<T\_y, T\_loc, T\_scale>

245  operands\_and\_partials(y, mu, sigma);

246

247  VectorView<const T\_y> y\_vec(y);

248  VectorView<const T\_loc> mu\_vec(mu);

249  VectorView<const T\_scale> sigma\_vec(sigma);

250  const double log\_half = std::log(0.5);

251  size\_t N = max\_size(y, mu, sigma);

252

253  for (size\_t n = 0; n < N; n++) {

254  const double y\_dbl = value\_of(y\_vec[n]);

255  const double mu\_dbl = value\_of(mu\_vec[n]);

256  const double sigma\_dbl = value\_of(sigma\_vec[n]);

257  const double scaled\_diff = (y\_dbl - mu\_dbl) / sigma\_dbl;

258  const double inv\_sigma = 1.0 / sigma\_dbl;

259  if(y\_dbl < mu\_dbl) {

260  // log cdf

261  cdf\_log += log\_half + scaled\_diff;

262

263  // gradients

264  if (!is\_constant\_struct<T\_y>::value)

265  operands\_and\_partials.d\_x1[n] += inv\_sigma;

266  if (!is\_constant\_struct<T\_loc>::value)

267  operands\_and\_partials.d\_x2[n] -= inv\_sigma;

268  if (!is\_constant\_struct<T\_scale>::value)

269  operands\_and\_partials.d\_x3[n] -= scaled\_diff \* inv\_sigma;

270  }

271  else {

272  //log cdf

273  cdf\_log += log(1.0 - 0.5 \* exp(-scaled\_diff));

274

275  //gradients

276  const double rep\_deriv = 1.0 / (2.0 \* exp(scaled\_diff) - 1.0);

277  if (!is\_constant\_struct<T\_y>::value)

278  operands\_and\_partials.d\_x1[n] += rep\_deriv \* inv\_sigma;

279  if (!is\_constant\_struct<T\_loc>::value)

280  operands\_and\_partials.d\_x2[n] -= rep\_deriv \* inv\_sigma;

281  if (!is\_constant\_struct<T\_scale>::value)

282  operands\_and\_partials.d\_x3[n] -= rep\_deriv \* scaled\_diff

283  \* inv\_sigma;

284  }

285  }

286  return operands\_and\_partials.to\_var(cdf\_log);

287  }

288

289  template <typename T\_y, typename T\_loc, typename T\_scale>

290  typename return\_type<T\_y,T\_loc,T\_scale>::type

291  double\_exponential\_ccdf\_log(const T\_y& y, const T\_loc& mu,

292  const T\_scale& sigma) {

293  static const char\* function

294  = "stan::prob::double\_exponential\_ccdf\_log(%1%)";

295

296  using stan::math::check\_finite;

297  using stan::math::check\_not\_nan;

298  using stan::math::check\_positive\_finite;

299  using stan::math::check\_consistent\_sizes;

300  using stan::math::value\_of;

301

302  double ccdf\_log(0.0);

303

304  // check if any vectors are zero length

305  if (!(stan::length(y)

306  && stan::length(mu)

307  && stan::length(sigma)))

308  return ccdf\_log;

309

310  check\_not\_nan(function, y, "Random variable", &ccdf\_log);

311  check\_finite(function, mu, "Location parameter", &ccdf\_log);

312  check\_positive\_finite(function, sigma, "Scale parameter", &ccdf\_log);

313  check\_consistent\_sizes(function, y, mu, sigma,

314  "Random variable", "Location parameter",

315  "Scale Parameter", &ccdf\_log);

316

317  using std::log;

318  using std::exp;

319

320  agrad::OperandsAndPartials<T\_y, T\_loc, T\_scale>

321  operands\_and\_partials(y, mu, sigma);

322

323  VectorView<const T\_y> y\_vec(y);

324  VectorView<const T\_loc> mu\_vec(mu);

325  VectorView<const T\_scale> sigma\_vec(sigma);

326  const double log\_half = std::log(0.5);

327  size\_t N = max\_size(y, mu, sigma);

328

329  for (size\_t n = 0; n < N; n++) {

330  const double y\_dbl = value\_of(y\_vec[n]);

331  const double mu\_dbl = value\_of(mu\_vec[n]);

332  const double sigma\_dbl = value\_of(sigma\_vec[n]);

333  const double scaled\_diff = (y\_dbl - mu\_dbl) / sigma\_dbl;

334  const double inv\_sigma = 1.0 / sigma\_dbl;

335  if(y\_dbl < mu\_dbl) {

336  //log ccdf

337  ccdf\_log += log(1.0 - 0.5 \* exp(scaled\_diff));

338

339  //gradients

340  const double rep\_deriv = 1.0 / (2.0 \* exp(-scaled\_diff) - 1.0);

341  if (!is\_constant\_struct<T\_y>::value)

342  operands\_and\_partials.d\_x1[n] -= rep\_deriv \* inv\_sigma;

343  if (!is\_constant\_struct<T\_loc>::value)

344  operands\_and\_partials.d\_x2[n] += rep\_deriv \* inv\_sigma;

345  if (!is\_constant\_struct<T\_scale>::value)

346  operands\_and\_partials.d\_x3[n] += rep\_deriv \* scaled\_diff

347  \* inv\_sigma;

348  }

349  else {

350  // log ccdf

351  ccdf\_log += log\_half - scaled\_diff;

352

353  // gradients

354  if (!is\_constant\_struct<T\_y>::value)

355  operands\_and\_partials.d\_x1[n] -= inv\_sigma;

356  if (!is\_constant\_struct<T\_loc>::value)

357  operands\_and\_partials.d\_x2[n] += inv\_sigma;

358  if (!is\_constant\_struct<T\_scale>::value)

359  operands\_and\_partials.d\_x3[n] += scaled\_diff \* inv\_sigma;

360  }

361  }

362  return operands\_and\_partials.to\_var(ccdf\_log);

363  }

364

365  template <class RNG>

366  inline double

367  double\_exponential\_rng(const double mu,

368  const double sigma,

369  RNG& rng) {

370  using boost::variate\_generator;

371  using boost::random::uniform\_01;

372  using std::log;

373  using std::abs;

374

375  static const char\* function

376  = "stan::prob::double\_exponential\_rng(%1%)";

377

378  using stan::math::check\_finite;

379  using stan::math::check\_positive\_finite;

380

381  check\_finite(function, mu, "Location parameter", (double\*)0);

382  check\_positive\_finite(function, sigma, "Scale parameter", (double\*)0);

383

384  variate\_generator<RNG&, uniform\_01<> >

385  rng\_unit\_01(rng, uniform\_01<>());

386  double a = 0;

387  double laplaceRN = rng\_unit\_01();

388  if(0.5 - laplaceRN > 0)

389  a = 1.0;

390  else if(0.5 - laplaceRN < 0)

391  a = -1.0;

392  return mu - sigma \* a \* log(1 - 2 \* abs(0.5 - laplaceRN));

393  }

394  }

395 }

396 #endif

stan::agrad::fabs

fvar< T > fabs(const fvar< T > &x)

**Definition:** fabs.hpp:18

traits.hpp

stan::agrad::OperandsAndPartials::to\_var

T\_return\_type to\_var(double logp)

**Definition:** partials\_vari.hpp:138

stan::math::check\_positive\_finite

bool check\_positive\_finite(const char \*function, const T\_y &y, const char \*name, T\_result \*result)

**Definition:** check\_positive\_finite.hpp:12

stan::math::sign

int sign(const T &z)

**Definition:** sign.hpp:9

error\_handling.hpp

stan::length

size\_t length(const T &)

**Definition:** traits.hpp:159

stan::prob::double\_exponential\_log

return\_type< T\_y, T\_loc, T\_scale >::type double\_exponential\_log(const T\_y &y, const T\_loc &mu, const T\_scale &sigma)

**Definition:** double\_exponential.hpp:24

stan::DoubleVectorView

DoubleVectorView allocates double values to be used as intermediate values.

**Definition:** traits.hpp:358

stan::math::check\_finite

bool check\_finite(const char \*function, const T\_y &y, const char \*name, T\_result \*result)

Checks if the variable y is finite.

**Definition:** check\_finite.hpp:52

partials\_vari.hpp

stan::agrad::value\_of

T value\_of(const fvar< T > &v)

Return the value of the specified variable.

**Definition:** value\_of.hpp:16

stan::prob::double\_exponential\_ccdf\_log

return\_type< T\_y, T\_loc, T\_scale >::type double\_exponential\_ccdf\_log(const T\_y &y, const T\_loc &mu, const T\_scale &sigma)

**Definition:** double\_exponential.hpp:291

constants.hpp

stan::is\_vector

**Definition:** traits.hpp:83

stan::agrad::OperandsAndPartials

A variable implementation that stores operands and derivatives with respect to the variable...

**Definition:** partials\_vari.hpp:76

traits.hpp

stan::return\_type::type

boost::math::tools::promote\_args< typename scalar\_type< T1 >::type, typename scalar\_type< T2 >::type, typename scalar\_type< T3 >::type, typename scalar\_type< T4 >::type, typename scalar\_type< T5 >::type, typename scalar\_type< T6 >::type >::type type

**Definition:** traits.hpp:406

stan::is\_constant\_struct

Metaprogram to determine if a type has a base scalar type that can be assigned to type double...

**Definition:** traits.hpp:57

stan::math::value\_of

double value\_of(const T x)

Return the value of the specified scalar argument converted to a double value.

**Definition:** value\_of.hpp:24

stan::prob::include\_summand

Template metaprogram to calculate whether a summand needs to be included in a proportional (log) prob...

**Definition:** traits.hpp:35

stan::agrad::OperandsAndPartials::d\_x2

VectorView< double \*, is\_vector< T2 >::value, is\_constant\_struct< T2 >::value > d\_x2

**Definition:** partials\_vari.hpp:83

stan::agrad::abs

fvar< T > abs(const fvar< T > &x)

**Definition:** abs.hpp:19

stan::prob::double\_exponential\_cdf

return\_type< T\_y, T\_loc, T\_scale >::type double\_exponential\_cdf(const T\_y &y, const T\_loc &mu, const T\_scale &sigma)

Calculates the double exponential cumulative density function.

**Definition:** double\_exponential.hpp:138

stan::math::check\_consistent\_sizes

bool check\_consistent\_sizes(const char \*function, const T1 &x1, const T2 &x2, const char \*name1, const char \*name2, T\_result \*result)

**Definition:** check\_consistent\_sizes.hpp:13

stan::max\_size

size\_t max\_size(const T1 &x1, const T2 &x2)

**Definition:** traits.hpp:191

stan::prob::double\_exponential\_cdf\_log

return\_type< T\_y, T\_loc, T\_scale >::type double\_exponential\_cdf\_log(const T\_y &y, const T\_loc &mu, const T\_scale &sigma)

**Definition:** double\_exponential.hpp:215

stan::prob::double\_exponential\_rng

double double\_exponential\_rng(const double mu, const double sigma, RNG &rng)

**Definition:** double\_exponential.hpp:367

stan::math::check\_not\_nan

bool check\_not\_nan(const char \*function, const T\_y &y, const char \*name, T\_result \*result)

Checks if the variable y is nan.

**Definition:** check\_not\_nan.hpp:56

sign.hpp

value\_of.hpp

stan::agrad::OperandsAndPartials::d\_x1

VectorView< double \*, is\_vector< T1 >::value, is\_constant\_struct< T1 >::value > d\_x1

**Definition:** partials\_vari.hpp:82

stan::agrad::OperandsAndPartials::d\_x3

VectorView< double \*, is\_vector< T3 >::value, is\_constant\_struct< T3 >::value > d\_x3

**Definition:** partials\_vari.hpp:84

stan::agrad::log

fvar< T > log(const fvar< T > &x)

**Definition:** log.hpp:15

stan::VectorView

VectorView is a template metaprogram that takes its argument and allows it to be used like a vector...

**Definition:** traits.hpp:275

stan::agrad::exp

fvar< T > exp(const fvar< T > &x)

**Definition:** exp.hpp:16


---

[ Stan Home Page ]

*© 2011–2014,
Stan Development Team.*
